# Recent advances in soluble decellularized extracellular matrix for heart tissue engineering and organ modeling

**DOI:** 10.1177/08853282231207216

**Published:** 2023-11-24

**Authors:** Golara Kafili, Hannaneh Kabir, Amirhossein Jalali Kandeloos, Elham Golafshan, Sara Ghasemi, Shohreh Mashayekhan, Nayere Taebnia

**Affiliations:** 1 Institute for Nanoscience and Nanotechnology, 68260 Sharif University of Technology, Tehran, Iran; 2 Molecular Cell Biomechanics Laboratory, Departments of Bioengineering and Mechanical Engineering, 1438 University of California, Berkeley, CA, USA; 3 Department of Printing Science and Technology, 185205 Institute for Colour Science and Technology, Tehran, Iran; 4 Department of Chemical and Petroleum Engineering, 68260 Sharif University of Technology, Tehran, Iran; 5 Department of Physiology and Pharmacology, 27106 Karolinska Institute, Stockholm, Sweden

**Keywords:** Cardiovascular tissue engineering, decellularized extracellular matrix, regenerative medicine, heart tissue modeling, biofabrication approaches

## Abstract

Despite the advent of tissue engineering (TE) for the remodeling, restoring, and replacing damaged cardiovascular tissues, the progress is hindered by the optimal mechanical and chemical properties required to induce cardiac tissue-specific cellular behaviors including migration, adhesion, proliferation, and differentiation. Cardiac extracellular matrix (ECM) consists of numerous structural and functional molecules and tissue-specific cells, therefore it plays an important role in stimulating cell proliferation and differentiation, guiding cell migration, and activating regulatory signaling pathways. With the improvement and modification of cell removal methods, decellularized ECM (dECM) preserves biochemical complexity, and bio-inductive properties of the native matrix and improves the process of generating functional tissue. In this review, we first provide an overview of the latest advancements in the utilization of dECM in *in vitro* model systems for disease and tissue modeling, as well as drug screening. Then, we explore the role of dECM-based biomaterials in cardiovascular regenerative medicine (RM), including both invasive and non-invasive methods. In the next step, we elucidate the engineering and material considerations in the preparation of dECM-based biomaterials, namely various decellularization techniques, dECM sources, modulation, characterizations, and fabrication approaches. Finally, we discuss the limitations and future directions in fabrication of dECM-based biomaterials for cardiovascular modeling, RM, and clinical translation.

## Graphical abstract

**Graphical Abstract:**
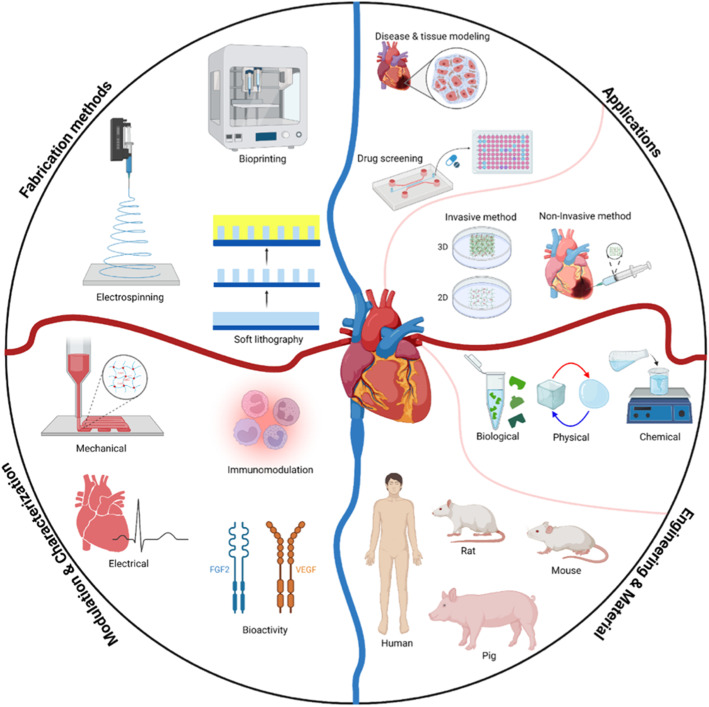

## Introduction

Cardiovascular diseases (CVDs), including myocardial infarction (MI) and coronary heart disease, are the leading cause of death in the world. CVDs caused an estimated 18 million deaths in 2020 which is responsible for 34% of all worldwide deaths,^1^ of which 85% were because of heart attack and stroke. In the United States, one person dies every 36s from CVDs and the disease costs $363 billion each year.^2,3^ Cardiomyocytes are not able to regenerate the damaged myocardium and lost cardiac muscle is replaced by scar tissue.^4^ Furthermore, 10% of drugs approved by the US Food and Drug Administration (FDA) have been discharged due to their cytotoxicity from 1975 to 2015.^5^ Currently, the common treatment for a damaged or failing heart is transplantation. However, a large number of patients need a healthy heart from a donor to save their lives, and heart donors are very limited worldwide to meet the demands.^6^

Tissue engineering (TE) plays a prominent role in addressing this challenge. TE aims to regenerate the tissue architecture and produce replicas to substitute damaged organs like the heart. Besides, recent progress in cell reprogramming has played a crucial role in the development of cardiovascular-engineered tissue.^7^ Along this vein, choosing suitable materials to mimic the native heart extracellular matrix (ECM) should not be underestimated. The biomaterials used for TE should have ideal mechanical and chemical properties to induce expected cellular behaviors including migration, adhesion, proliferation, and differentiation.^8^ To date, synthetic polymers, natural biomaterials, and a combination of the two have been used in cardiac TE. Poly (Ethylene Glycol) PEG, Poly (Caprolactone) PCL, Poly (Glycolic acid) PGA, and Poly (DL-Lactic-co-Glycolic) PLGA are common synthetic polymers used for this purpose. They present high mechanical strength and tunable properties due to the functional groups present in their structure. However, these synthetic biomaterials lack cell-binding sites and should be post-treated to obtain the required motifs.^9^ Naturally derived biomaterials such as collagen I and gelatin have also been widely applied in cardiac TE due to their biocompatibility, hydrophilicity, and inclination to promote cellular attachment and proliferation.^10^ Since the composition of natural proteins in different ECMs varies according to the tissue function, decellularized cardiac tissue makes a promising candidate for cardiac TE among other natural biomaterials.^8^

Decellularized extracellular matrix (dECM) is the most attractive material given its ability to maintain essential properties of the native tissue. Cardiac dECM provides a proper and biomimetic circumstance for myocardiocytes to grow and regenerate damaged heart tissue.^11^ The decellularization technique has been exploited since 1940. William Poel (1948) was a pioneer who produced acellular homogenate from muscles by pulverization method at −70°C, where pulverized and thawed tissues were homogenized in water with a cylinder and rotating plunger.^12^ Decellularization of cardiac tissue has progressed swiftly in the recent decade.^13^ Cardiac dECM has been often derived from porcine, murine, and human sources. Ott and his colleagues decellularized the whole rat heart for the first time with a specific solution through the coronary perfusion chamber to keep the heart geometry and vascular architecture intact. They generated the heart’s physiological function to some extent by culturing the cardiac and endothelial cells with a perfusion bioreactor culture condition.^14^ Using the same method, Wainwright and his coworkers developed acellular porcine whole heart ECM and demonstrated that the obtained dECM resembled the native ECM in terms of composition, which containes collagen, glycosaminoglycan, and elastin. In addition, it possessed adequate mechanical strength to support the differentiation and spatial organization of reseeded stem cells.^15^ Indeed, human decellularized cardiac tissue is more favorable than porcine and murine sources, however, this technique is hindered by the scarcity of donor organs, especially the heart. Sanchez et al. developed the human cardiac ECM with the preserved geometry and vascular architecture for the first time and demonstrated that it could successfully support cardiac gene expression and electrical coupling when it was reseeded with parenchymal cells.^16^

Decellularization approaches are either mechanical or chemical. Mechanical strategies include freeze-thawing or hydrostatic pressure to remove cells and genetic materials. Although these methods are advantageous in retaining biomechanical and biochemical properties, they can develop immunogenicity due to the incomplete removal of genetic materials.^17^ Chemical approaches apply surfactants, acids, and bases to lyse cells through disarranging the phospholipid cell membranes. Although these reagents thoroughly remove unwanted materials, they can damage structural and signaling proteins required for cell regulations.^18^ Given the above-mentioned pros and cons associated with each method, the combination of chemical and mechanical approaches has been employed to retain desired tissue characteristics.^19^ When the dECM is achieved, different types of stem cells such as embryonic stem cells, mesenchymal stem cells (MSCs), and induced pluripotent stem cells (iPSCs) are cultured on the acellular tissue to induce spontaneous tissue repair.^13,20–22^

This review aims at presenting an overview of the great potential of cardiac dECM which serves as a naturally-derived platform for cardiac TE and organ modeling. First, the application of dECM for *in vitro* disease modeling and drug screening is discussed, and then recent progress in dECM-based constructs in tissue regeneration is presented. In the next step, engineering and material considerations including modulations, characterizations, and fabrication methods is elucidated. Finally, future perspectives of dECM strategy for regenerative medicine applications are illustrated.

## dECM for *in vitro* model systems

### Disease and tissue modeling

**Figure 1.**
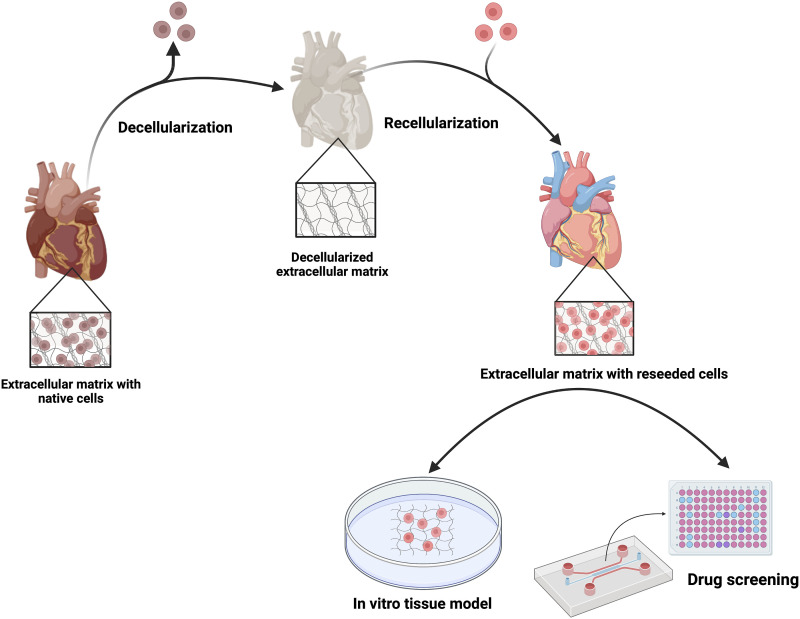
Schematic illustration of the steps required for the preparation of dECM-based in vitro tissue models for drug screening applications.

Different diseases causing abnormality in the structure or function of the myocardium are called cardiomyopathy and lead to heart failure.^23^ Cardiomyopathies can be classified into main categories of hypertrophic cardiomyopathy (HCM), dilated cardiomyopathy (DCM), restrictive cardiomyopathy (RCM), and arrhythmogenic right ventricular cardiomyopathy (ARVC). The pathophysiological mechanisms of such diseases are not fully understood and require developing authentic disease models.^24^ To date, various types of models have been developed to advance our understanding of cardiomyopathies, including animal models, 2D and 3D *in vitro* cellular models such as cardiac spheroids or cell sheets, engineered cardiac tissues (ECTs) which combine biomaterials and 3D scaffolds with living cells, and microfluidic devices to develop biomimetic tissue models.^25^ Recellularization of decellularized heart tissues has opened a new avenue in the pursuit of *in vivo*-like and compatible ECTs for cardiac model development.^26^ Owing to their high biocompatibility and physiological relevance in terms of chemical composition and biomechanical properties, they serve as ideal scaffolds and have been rapidly picked up by researchers in tissue models for drug screening applications (Figure 1).

**Figure 2.**
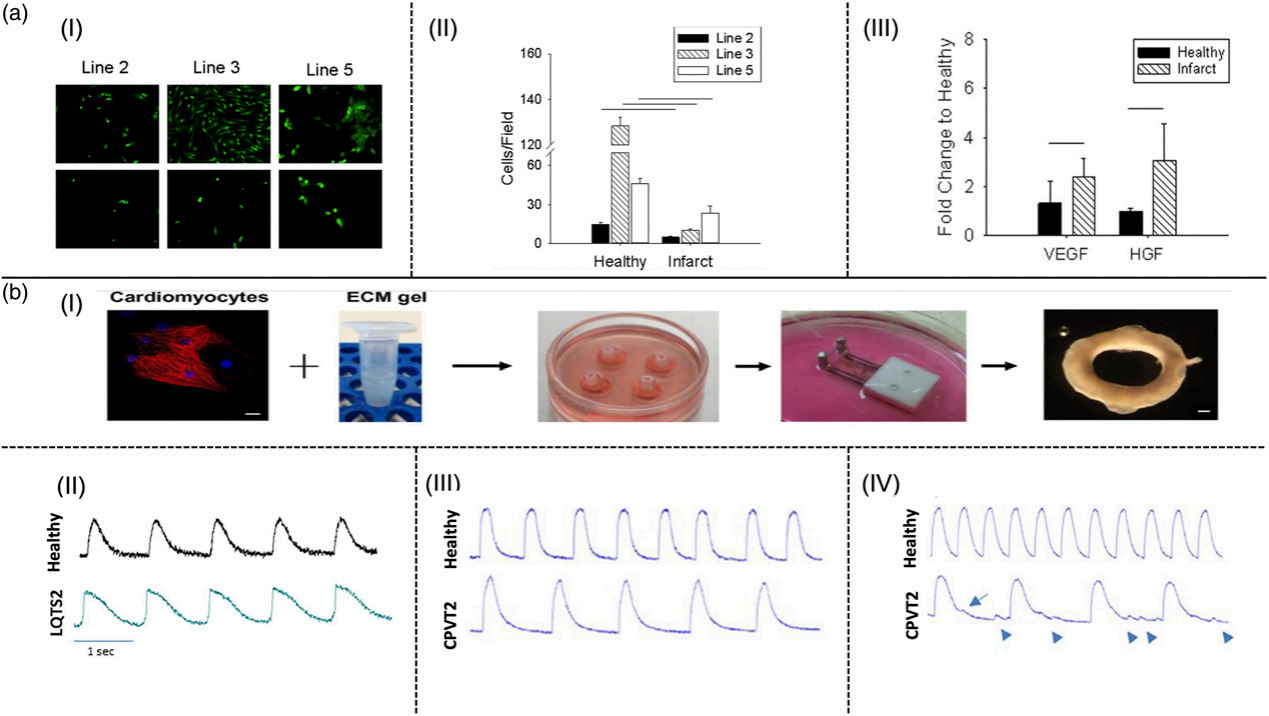
(a) ECT model based on cECM combined with polyacrylamide for predicting stem cell fate after in vivo injection,^30^ (I) Impeding cell proliferation after 5 days culture on infarct model compared to healthy model for all three lines, (II) Quantitative analysis of the image shows rapid cell proliferation on the healthy environment compared to infarct model, (III) Increased expression of pro-survival (HGF) and pro-angiogenesis (VEGF) growth factors within infarct model relative to the healthy model; (b-I) The fabrication process of ECT based on cECM-chitosan containing hiPSC-CMs (scale bar at the left panel: 20 µm, scale bar at the right panel: 0.5 mm),^31^ (II) Line scan images depicting prolongation of AP of hiPSC-CMs inside LQTS-ECT model compared to healthy ECT model, (III) Laser confocal Ca^2+^ imaging of hiPSC-CMs at baseline in CPVT-ECT model relative to the healthy-ECT model, (IV) Irregular activity of hiPSC-CMs in laser confocal Ca^2+^ imaging in CPVT-ECT model (arrows) compared to healthy ECT model after addition of 10 µM isoproterenol.

To cite an interesting example, decellularized rat hearts cultured with various types of human cells, including human embryonic kidney cells (HEK293), cardiac fibroblasts, and cardiac progenitor cells derived from iPSCs, has been reported to support cell attachment and spreading into the tissue.^27^ Furthermore, it has been demonstrated that perfusion of growth factors facilitates the reformation process of heart muscles and re-endothelialization.^21^ Jung et al. have used decellularized swine aorta coupled with a heart circulatory and beating mimicry system as a model for screening the vessel adhesives. The decellularized aorta has shown no significant difference in terms of maximum load-bearing, tensile strain, and modulus compared to the original vessel when flow pressure is applied, even after the treatment of a 1 cm length rupture with cyanoacrylate adhesive. These results prove the efficacy of the decellularized matrix to serve as a perfect model system.^28^ Hogan et al. have wrapped a fibrin patch containing active cardiomyocyte cells around decellularized adult rat hearts to fabricate a functional bioartificial heart muscle model.^29^ Their results show that after 1–2 days of culture, the construct was beating normally with a contractile rate of 4.5 Hz. Sullivan and colleagues have designed an *in vitro* model for myocardial infarction using hybrid dECM isolated from both infarcted and healthy rat hearts combined with polyacrylamide gels.^30^ These models were used to evaluate the fate of cardiac progenitor cells (CPCs) for *in vivo* transplantation into the MI region as a clinical cell therapy method. The model predicts that the infarct environment attenuates the potential of c-Kit+ CPCs for cardiac and vascular differentiation and inhibits cell adhesion and proliferation (Figure 2(A-I) and (A-II)). This is consistent with other reports that state the signaling moieties of native myocardium are not able to induce spontaneous differentiation of c-Kit+ CPCs.^32^ However, pro-survival and pro-angiogenic paracrine signaling of CPCs with host cells, which leads to their functional repair capacity was seen in clinical experiments (Figure 2(A-III)).^30^ In another work, cardiac extracellular matrix (cECM)-derived hydrogel containing chitosan and human-induced pluripotent stem cell-derived cardiomyocytes (hiPSC-CMs) have been developed as an ECT model (Figure 2(B-I)).^31^ To model familial arrhythmogenic syndrome conditions, hiPSC-CMs were derived from patients with two types of inherited arrhythmogenic disorders named long QT syndrome type 2 (LQTS2) and catecholaminergic polymorphic ventricular tachycardia type 2 (CPVT2). The results demonstrate longer action potential duration (APD) in LQTS-ECTs compared to healthy control ECTs (Figure 2(B-II)). On the other hand, irregularity in Ca^2+^ transient and arrhythmias was observed in 19.1% of CPVT-ECTs (Figure 2(B-III) and (B-IV)). Interestingly, the arrhythmogenic activity in deceased ECT models was lower than in single-cell models, which stems from the interactions between connected CMs in multicellular ECT models.^31^

### Drug screening

New drug development requires animal testing and clinical research to evaluate its safety and therapeutic efficacy. However, one significant challenge in the process of developing drugs is the use of animal models, since they are substantially different in genetic backgrounds and cardiac physiology compared to humans.^33–35^ For instance, some drugs were found ineffective or toxic in animal models in the specified dosing therapy, whereas they are effective and safe for humans, and such differences in drug responses often hinder the research and development path.^36^ Therefore, it is crucial to develop predictive and physiologically relevant preclinical models to recapitulate the tissue and disease phenotypes for screening novel drugs.

With the emergence of hiPSC, patient-derived *in vitro* platforms have been established to model different diseases and assess drugs.^33,37^ To understand the effect of specific compounds on the cardiovascular system, different functional parameters such as electrophysiological and calcium handling properties, contraction rate, and contractility force should be investigated.^35,38^ One of the limitations of the produced hiPSC-CMs is functional immaturity which restricts their utilization in *in vitro* heart disease modeling and drug screening.^33,39^ To address this shortcoming, various strategies have been employed^40,41^ such as introducing mechanical and electrical stimulation,^42^ co-culture systems,^43^ and 3D culturing approaches which enhance hiPSC-CM maturation and the functionality of engineered platforms. Several studies have emulated the native tissue microenvironment to highlight the effect of the 3D environment on the maturation of hiPCS-CM.^44,45^ Various kinds of 3D scaffolds have been utilized for personalized disease modeling and drug screening applications, among which, dECM-based scaffolds represent one of the most suitable approaches. Blazeski et al. repopulated porcine dECM by hiPSC-CMs to produce engineered heart slices (EHS), which exhibited morphological and practical enhancement over traditional culture conditions. EHS demonstrated good anisotropy, positive inotropic responses to isoproterenol, and long-term electrophysiological activity.^46^ Schwan et al. repopulated laser-cut sheets of decellularized myocardium with neonatal rat ventricular myocytes to produce engineered heart tissues (EHTs) with tunable biomechanical properties. EHTs beat synchronously on the fifth day and demonstrated robust activity until the 21st day. The direction of the fibers in the scaffold affected the peak twitch stress, which indicated the practical efficiency of this system in guiding the cells toward their natural contraction. The anisotropy of the scaffolds enables the study of the effect of fiber angles on cellular stretch and response to the created tension. Unlike off-axis stretches, stretches in the fiber axis direction were found to increase the expression of brain natriuretic peptides (BNPs). In this technique, cardiac muscle cells derived from fetal stem cells and hiPSCs were used to provide robust EHTs. In conclusion, EHTs could act as a viable platform for novel mechano-transduction experiments and characterizing the biomechanical function of patient-derived CMs.^47^

Goldfracht and collaborators^31^ produced engineered heart tissue by combining patient-specific and healthy hiPSC-CMs with chitosan-dECM hydrogel to evaluate the ability of this platform for disease modeling and drug screening. They examined the effect of various pharmacological agents (isoproterenol, carbamylcholine, E-4031, ATX2) on the electrophysiological and calcium-handling characteristics of the engineered heart tissue. Their results revealed that the scaffold can enhance the degree of maturation in hiPSC-CMs at the single-cell level, and exhibit electrophysiological properties of the engineered cardiac construct for drug efficiency testing and personalized therapy. In a similar study, to improve the phenotypic maturity of hiPSCs, Tsui et al.^48^ developed a 3D-printed bioactive and electroconductive construct via a combination of reduced graphene oxide (rGO) with dECM for drug cardiotoxicity evaluation. They modulated the mechanical and electrical properties of the hybrid bioink by altering the rGO content and its degree of reduction. In this work, the effect of Cisapride doses on the electrophysiological function of bioprinted tissue was evaluated. The result demonstrated the capability of this engineered construct in producing physiologically relevant drug responses. Similarly, Almeida et al. combined dECM particles with hiPSCs to enhance their maturity for *in vitro* model application. Their result confirms that the presence of dECM in hiPSC-CM aggregates improves the phenotypical and structural maturation of cardiomyocytes as well as their calcium handling compared to pure hiPSC-CM aggregates.^45^

## dECM in regenerative medicine

### Invasive methods (prefabricated 2D and 3D scaffolds)

This section focuses on the application of prefabricated 2D and 3D dECM scaffolds in RM which are dominantly transplanted through invasive methods such as surgery. Before *in vivo* and clinical trials, engineering of pre-fabricated decellularized scaffolds includes three main steps: (1) Decellularization of the tissue to form a scaffold, (2) cell seeding on the scaffold, and (3) *in vitro* culture conditioning, such as bioreactor conditioning.^49–51^ ECM has an impact on both cell and organ development.^45,52–58^ Several studies have shown that 2D culturing of various cell types (e.g., cardiac progenitor cells on porcine cardiac ECM can affect rat and human cardiomyocyte (CM) growth and maturation.^59–62^

*In situ* recellularization of dECM is possible through the chemical modification of decellularized tissues. In a study by Ota et al., a fibronectin-hepatocyte growth factor was introduced into the decellularized porcine aortic valves to assist the attachment and proliferation of endogenous cells.^63^ In another study, to fabricate self-seeding heart valves with quick maturation *in vivo*, Jordan et al. conjugated CD133 antibodies to porcine pulmonary valve dECM scaffolds to induce the formation of endothelial tissue via taking CD133-positive endothelial progenitor cells from the flow. Recently, Li et al. linked poly ε-caprolactone (PCL) nanoparticles containing osteoprotegerin (OPG) to a decellularized valve and constructed a novel composite with an anti-calcification function and a constant release utility. The composite was implanted and after 2 months, the valve attained an almost completed morphological shape.^64^

One of the primary challenges of engineering dECM-based scaffolds is the development of a physiologically proper cell population inside the tissue. Dai et al. produced a hybrid scaffold by combining a porous matrix metalloproteinase (MMP), poly (ethylene glycol) (PEG) hydrogel containing stromal cell-derived factor-1α (SDF-1α), and a decellularized porcine aortic valve as a mechanical support. The combinatorial scaffold improved the viability, adhesion, and proliferation of bone marrow mesenchymal stem cells (BMSCs). Moreover, it aided their differentiation into valve interstitial-like cells. This approach also protected the scaffold from thrombosis.^65^In another study by Pok et al., a cell-free porcine-derived dECM was implanted in the right ventricle of a rat to repair connective tissue. After 16 weeks of treatment with the patch, the function of the right ventricular in the rat model returned to normal.^66^

Fabrication of cell-seeded scaffold-based cardiac patches can overcome the limitations of cell therapy such as low retention and engraftment of transplanted cells. However, these patches also suffer from limitations; one of which is their short shelf-life. In other words, they must be used immediately after preparation. Immunogenicity and the high cost in terms of labor and material are the other two main concerns when producing cellular cardiac patches. Considering this, Shah et al. examined the therapeutic consequences of using a decellularized porcine myocardium slices (dPMS) cell-free patch in a rat severe MI model. dPMSs with various thicknesses were grafted to the infarcted area, and their impact on cardiac function and host interactions was evaluated. The results showed that the patches made by decellularized porcine myocardium were very well placed and, exhibited remarkable growth and development. More interestingly, they prevented atrophy of the left ventricle after MI and the host cells were successfully adapted to the implanted patch, infiltrated, and started to grow and proliferate. Furthermore, angiogenesis was observed in their histological analysis.^67^ Huang and coworkers created an off-the-shelf cardiac patch consisting of porcine dECM and synthetic cardiac stromal cells (synCSCs). SynCSCs were fabricated by encapsulating human CSC-secreted factors into degradable polylactic-co-glycolic acid (PLGA) particles. They used the combination of 3D myoECM and synCSCs to complement each other as the former offers mechanical support and the latter secretes recreating factors. All constituents were cell-free to overcome the limitations of employing living cells and to provide a ready-to-use patch with a prolonged shelf life. The product preserved its potency after cryopreservation, promoted angiogenesis, enhanced cardiac function, and diminished the infarct size after patching on rat and pig hearts post-MI.^68^

Fong et al. compared the impact of 2D versus 3D, and fetal versus adult ECM on cellular behavior and development. They demonstrated that the iPSC-CMs development is improved when cells are incorporated within a 3D scaffold instead of a 2D culture. 3D scaffold aided the expression of calcium-handling genes. They also found that iPSC-CMs demonstrate improved calcium signaling and rate when cultured on 3D adult cardiac ECM. Moreover, 3D cultured cells were more responsive to caffeine, denoting enhanced accessibility to Ca in the sarcoplasmic reticulum. The results collectively indicate the important effect of both the dECM source and geometry on the fate of seeded cells.^69^

Based on these studies, prefabricated dECM scaffolds serve as powerful platforms for the development of functional cardiac tissues. However, they do suffer from some limitations. They need human or animal tissue sources, which are restricted by the finite supply and require cryopreservation for storage. Furthermore, given their animal or human sources, there always exist variations and inter-individual variability.^70^ Additionally, the existence of the ∝-gal epitope causing hyper-acute immune rejection is a great barrier to clinical xenotransplantation.^71,72^ Various chemical and enzymatic methods are used to denature the ∝-gal epitope,^73^ although particular challenges still hamper this path. Preservation of the desirable bioactivity of the ligands and maintenance of the recellularization capacity of the scaffold is another issue that needs further investigation. Surgical risk evaluations are mandatory before implementation in patients since cardiac patches might induce arrhythmia exacerbating normal cardiac function.^74^ All in all, developing a therapeutic pre-fabricated dECM scaffold necessitate a large evaluation of a miscellaneous group of functional results ranging from electrophysiology to contractile function.

### Non-invasive methods (injectable hydrogels)

Because of the limited self-renewal potential and proliferation of cardiac cells, promising strategies based on cell and protein therapy have been recently developed and investigated to improve post-MI heart function and prevent heart failure after infarction.^75^ Several studies have shown that the injection of stem cells into the heart wall could promote myocardial repair and inhibit scar formation.^76^ However, this method suffers from low post-translational cell survival and engraftment to the host cells of the injured site.^75^ To address these limitations, exploiting suitable scaffolds is an effective strategy to shelter the stem cells and enhance their survival while controlling their fate.^77^ Among different types of reported scaffolds, hydrogel-based biomaterials represent one of the most promising candidates for stem cell transplantation due to their capacity of retaining the cells in the infarcted site within a biomimetic microenvironment.^78^ This section focuses on *in-situ* forming injectable dECM-based hydrogels as a minimally invasive treatment for the regeneration and repair of cardiac tissue. Compared to the surgical methods, injectable hydrogels are delivered less invasively, and subsequently, reduce the injury to the adjacent tissues.^79^

Dynamic reciprocity among the biomaterial and the occupant cells is the most important advantage of ECM-based scaffolds over synthetic ones.^80,81^ Previous studies indicated that dECM-based hydrogels can promote tissue repair by inducing angiogenesis.^82,83^ Singelyn and coworkers reported that the dECM injection could improve angiogenesis and CMs survival in the infarcted zone. The main characteristics of the dECM that contribute towards tissue regeneration and differentiation include timely degradation and the capability of bioactive molecules release.^83^ Seif-Naraghi et al. showed the applicability of pericardial ECM as an autologous scaffold in rat models.^84^ In another study from the same group, they injected a porcine myocardial dECM-derived hydrogel in both pig and rat models 2 weeks post-MI. Results indicated that the dECM hydrogel improved cardiac function and increased ventricular volumes.^85^ Qiao et.al investigated the synergistic effects of the combination of adipose-derived stem cells (ADSCs) with dECM for MI treatment. Results demonstrated that the encapsulation of ADSCs into myocardial dECM is an effective strategy to reduce fibrosis and improve cardiac function.^86^

Selective and fast gelation under physiological conditions is a prerequisite for *in situ* formations of injectable hydrogels.^87^ dECM-based hydrogels can be easily injected into the myocardium at room temperature thanks to their thermo-responsive behavior. dECM solutions are mostly liquid at temperatures lower than 22°C and transform into gel at 37°C.^88^ The dECM hydrogels are highly viscoelastic and exhibit excellent cell viability and functionality for 3D cell encapsulation. These properties make them ideal for catheter injection. However, the majority of injectable dECM-based hydrogels still suffer from inferior mechanical properties, long gelation time, poor electrical conductivity, and rapid degradation rate.^89^ To overcome these obstacles, novel strategies based on hybrid or composite hydrogels have been recently reported. To this end, cardiac dECM hydrogels have been combined with silk, alginate, and chitosan.^90–93^ As an example, Curely et al. combined dECM with two types of alginate-based hydrogels with different blocks (high G block and high M block). They demonstrated that the high G block/dECM hybrid hydrogel has suitable rheological and mechanical properties and can be delivered using catheter injection.^90^ Stoppel et al. developed injectable dECM/silk fibroin hydrogel. Silk hydrogel was able to modulate the mechanical properties by free-radical crosslinking of tyrosines inside silk fibroin and porcine left ventricular dECM. Their result revealed that the presence of dECM hydrogel improves cell infiltration and vascularization.^91^ Efraim and coworkers used genipin or a combination of genipin and chitosan to regulate the mechanical characteristics of injectable dECM hydrogels. The addition of chitosan significantly increased Young’s moduli of the hydrogels. The bio-hybrid hydrogels were naturally remodeled by MSCs, supported cell viability, and positively affected the morphology and organization of cells.^92^ To overcome the poor electrical conductivity of the ECM-derived hydrogels which is important for cardiac tissue engineering,^94^ Musavi et al. developed an *in situ* forming hydrogel based on oxidized alginate (OA) and cardiac dECM containing 3-(2-aminoethyl amino) propyltrimethoxysilane-modified reduced graphene oxide (APTMS-rGO) which improved both mechanical characteristics and electrical conductivity of the hydrogel. Chemical cross-linking through Schiff’s base reaction of OA with amino groups of dECM and APTMS as well as ionic cross-linking of OA via calcium ions was employed to prepare a double network hydrogel with enhanced mechanical properties. Moreover, the presence of APTMS-rGO contributed towards better electrical conductivity of the polymeric network and induced cellular function and differentiation.^93^

Altogether, injectable dECM-based hydrogels with the integration of suitable cell sources are an advanced regenerative method that has been under development for clinical trials. These hydrogels can be injected with minimal invasiveness into target sites and adopt the irregular shapes of the native tissue. However, maintaining and improving the mechanical integrity of the injectable scaffolds is a challenging requirement that must be met to fabricate 3D hydrogel structures with improved mechanical properties.

## Engineering and materials considerations

### Decellularization method

Decellularization treatment is a necessary step in the preparation of dECM-based materials to eliminate the risk of potential immunogenicity and inflammation.^95^ However, the decellularization parameters should be optimized to remove cells (lower than the minimum criteria of 5 ng per µg of dry tissue) and to maintain the ECM architecture in terms of the structural proteins and glycosaminoglycans (GAGs).^96^ Furthermore, the residues of decellularization agents should not remain in the matrix after the treatment, because these compounds tend to induce cytotoxicity and prevent the cell repopulation process.^97^

To decellularize heart tissues for TE and RM applications, different groups of materials including detergents, chelating agents, and enzymatic agents have been used. In addition to these chemical methods, physical methods such as alternative freeze-thawing steps, agitation, and osmotic shocks facilitate the disruption of cellular membranes. The application of nucleases to remove any residues of nucleic acids decreases the immunogenic response to the decellularized organs or tissues.^98^ The most widely used decellularization agents for heart tissue are summarized in Table 1.

**Table 1.** Most useful decellularization agents for the heart.

Decellularization agent	Type of material	Advantage	Disadvantage
Triton X-100	Non-ionic detergent	Disruption of protein-protein interactions/breaking lipid-lipid and lipid-protein interactions	Alteration to ECM network
Sodium deoxycholate (SD)	Anionic detergent	Solubilization of cells and nucleic membrane/disruption of protein-protein interactions	Denaturation of proteins
Sodium dodecyl sulfate (SDS)	Cationic detergent	Solubilization of cellular membranes/disruption of protein-protein interactions	Aggressive denaturation of proteins/detrimental for ECM/damage to GAG moiety
Trypsin	Enzyme	Disruption of protein-protein interactions/dissociation of cells from their niche	Damage to ECM proteins/loss of bioactivity for collagen and elastin
Nuclease	Enzyme	Breaking nucleic acids into smaller pieces to facilitate their removal	Adherence to proteins of ECM
Ethylenediaminetetraacetic acid (EDTA)	Chelating agent	Separation of metal ions for dissociation of cells	Not efficient solely
Glycerol	Alcohol	Lysis of cells/removal of lipids	-
Ethanol	Alcohol	Lysis of cells/removal of lipids	Deterioration of ECM structure
Peracetic acid	Acid	Hydrolytic degradation of nucleic acids/Solubilization of cytoplasmic components	Disruption of ECM
Ammonium hydroxide	Alkaline	Hydrolytic degradation of biomolecules	Elimination of growth factors
NaCl	Hypotonic solution	Detachment of DNA from proteins	-
Tris-HCl	Hypertonic solution	Lysis of cells by osmotic shock	-

Results of a pioneering work by Ott et al. showed that decellularization with 1% SDS performs better than 1% Triton X-100 and 1% PEG, where perfusion of 1% SDS over the cannulated aorta in a modified Langerdorff system led to a fully decellularized heart construct, with a DNA content of below 4% of the native tissue and intact GAG content.^14^ Although Triton X-100 causes less damage to ECM structure, it is not as effective as SDS in cell removal.^99^ In another study, limited exposure to protease inhibitors in combination with 0.5% SDS, followed by 1% Triton X-100 treatment was investigated as a conservative decellularization protocol for the whole heart organ to assist the removal of SDS remains and nuclease digestion in perfusion/submersion conditions. This method has demonstrated good potential in the preservation of ECM integrity, the bioactivity of the organ, and the coronary arterial tree.^97^ It has been reported that Triton X-100/sodium deoxycholate (SD) combinations can be considered an effective cell removal protocol without compromising ECM architecture and mechanical properties. A combination of 1% Triton X-100 with 0.5, 1, and 2% SD treatments have shown perfect decellularization of porcine aortic heart valves as reported by Luo et al.^100^

Although combinations of SDS and Triton X-100 are effective in the removal of cell nuclei, they may lead to the denaturation of ECM proteins and subsequent decrease of mechanical properties.^101^ Seo et al. have proposed a novel detergent-free decellularization method based on supercritical carbon dioxide and ethanol (scCO_2_-EtOH) co-solvent system to overcome this limitation.^101^ CO_2_ can be considered as a non-flammable and non-toxic gas and scCO_2_ has been applied for the sterilization of scaffolds and decellularization of native organs.^102–104^ In this system, the EtOH solubilizes the cellular membranes, followed by cellular component removal by penetration of scCO_2_ into the tissue. This method prevents the disruption of ECM proteins and contributes to the treatment of ischemic disease by preserving angiogenic factors including vascular endothelial growth factor (VEGF), fibroblast growth factor (FGF), and platelet-derived growth factor (PDGF) present in the ECM of the heart which eventually promotes the angiogenesis.^101^ It is noteworthy that the remaining double-stranded of the detergent-treated heart tissue was less than that of scCO_2_-EtOH-treated one. On the other hand, the scCO_2_-EtOH system performed much better in the preservation of ECM proteins, GAGs, and GFs. Therefore, further investigations on the scCO_2_-EtOH decellularization system with the main focus on the improvement of the DNA removal capacity could add more value to this promising method.

Another approach for decellularization of the heart organ is the trypsin enzyme combined with chelating agents or detergents, which is most effective at 37°C and short incubation times.^105^ Decellularization of the heart with trypsin enzyme solely leads to partial removal of cells and aggressive deterioration of ECM structure, which limits the biochemical activity of the obtained scaffold.^106^ On the other hand, it has been demonstrated that using the combination of trypsin and detergents is less effective than detergent cocktails in terms of cell removal and preservation of structural properties.^107^ As a result, trypsin is not considered an efficient decellularization agent for heart tissue, neither alone nor in combination with detergents.^97^

Most recently, a vacuum-assisted detergent-enzymatic decellularization method has been applied to heart valves.^108^ Vacuum as a physical factor can increase the diffusion rate of decellularization agents into the tissues, which reduces the decellularization process time.^109^ Results of this study revealed that shorter exposure times to the SD detergent in the vacuum-assisted method cause lower damage to the collagen fibers and their directional alignment, and the integrity of the ECM network, is preserved. Better still, using a vacuum improves the DNA removal efficiency, but the loss of elastin and water-soluble GAGs is inevitable during decellularization procedures.^108^

**Figure 3.**
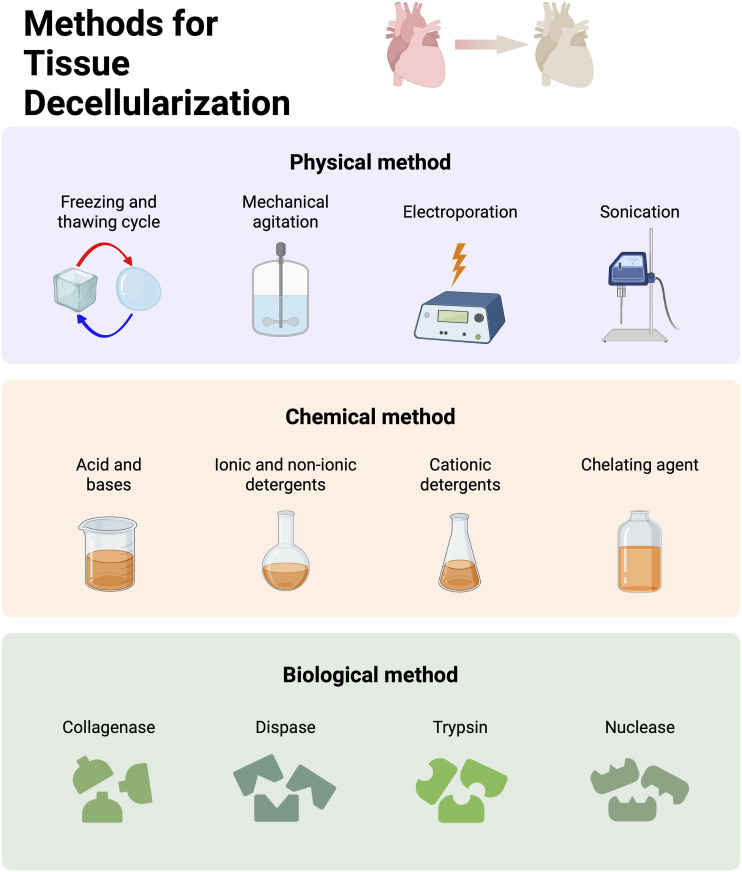
Schematic demonstrates various physical, chemical, and biological methods that can be used for tissue decelluraization.

N-Lauroylsarcosine sodium salt is another novel compound that has been investigated for the decellularization of heart valves. The heart valves treated with this biocompatible aminoacid-derived detergent (at 1 %w/v) under a vacuum atmosphere (‹100 Pa) represented no obvious cell nuclei after decellularization.^110^ However, treatment of heart valves with this compound causes the loss of GAGs and structural proteins which is unavoidable.^110^ Various physical, chemical, and biological methods that can be employed for decellularization of tissues are depicted in Figure 3.

### Source of the dECM

**Figure 4.**
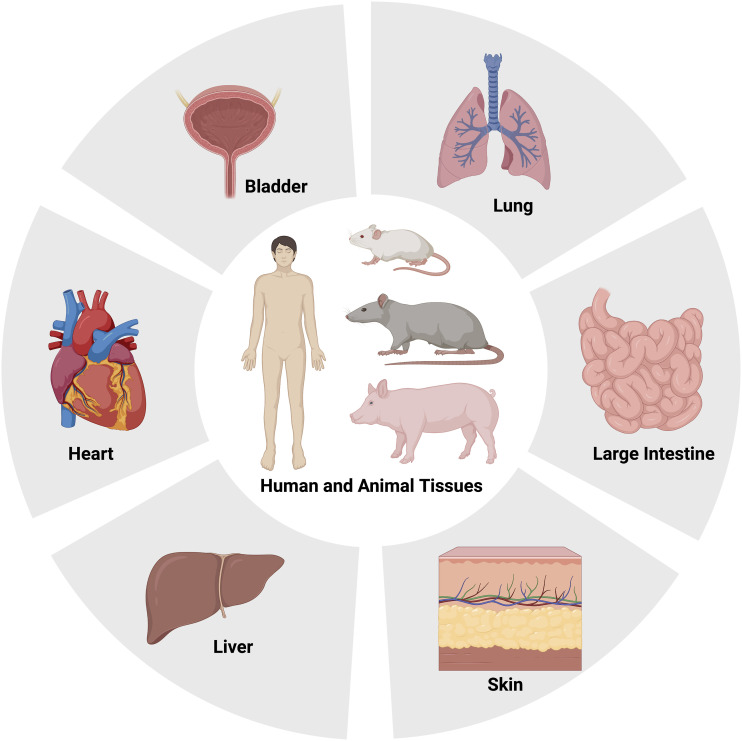
Different dECM sources based on the classification of species and target tissues.

The dECM source can be classified based on the species and target tissues (Figure 4). dECM obtained from myocardial tissues has been extensively utilized in cardiac regeneration thanks to the variety of benefits and advantages it offers. Upon proper decellularization, the cardiac dECM scaffold can retain biochemical and mechanical cues from the native tissue that aids cell adhesion, proliferation, and cardiovascular differentiation throughout subsequent recellularization.^98,111^ Cardiac dECM slices/scaffolds derived from a variety of species (e.g., rat, mouse, pig, and human) have been employed for cardiac tissue engineering and demonstrated promising results.^112–115^ Several studies have shown that rat cardiac dECM scaffolds can enhance the attachment, survival, growth, differentiation, and maturation of hiPSCs, and support the viability, proliferation, and migration of cardiac progenitor cells in addition to neonatal CMs.^113,114,116,117^ To cite an example, Wang et al. reseeded rat cardiac dECM scaffolds with iPSC-derived cardiac cells. Upon grafting on the severe rat MI model, the recellularized cardiac dECM has been demonstrated to reduce the infarct size, improve the thickness of the wall and assist vascularization.^114^ Porcine myocardium dECM reseeded with neonatal rat ventricular cells has also been reported to promote cell elongation, alignment, and synchronous beating, resulting in the formation of anisotropic functional tissue.^118^ Decellularized porcine myocardium patch after implantation on rat MI models, has been demonstrated to induce vascularization and ECM remodeling denoted by enhanced M2/M1 macrophage phenotypic proportion.^119^ More interestingly, both rat and porcine cardiac dECM exhibited greater stiffness in comparison to the freshly dissected (control) native myocardium tissue, indicating that the presence of cells does not have a significant impact on the stiffness of the cardiac tissue.^117,120^ Human cardiac dECM scaffold has also been investigated and improved cell adhesion, survival, and maturation were observed for human umbilical cord blood-derived MSCs, murine iPSC-derived CMs, murine neonatal CMs, human cardiac progenitor cells (hCPCs), iPSC-CMs, and human cardiac primitive cells.^16,20,121,122^ In comparison to MSCs, iPSC-CMs showed less cell adhesion, proliferation, and infiltration on the human dECM slices after implantation.^121^ Sanchez et al. reseeded a human dECM scaffold with hCPCs. The results have shown an improvement in the expression of cardiac markers such as bMHC, MEF2C, Nkx2.5, and TnnT, when compared to conventional 2D cultures. After the culture of HUVECs on dECM slices, an endocardium lining and vasculature were formed. The CMs were arranged into nascent muscle bundles and demonstrated mature calcium kinetics and electrical coupling on the dECM scaffold.^16^

In addition to primary cardiac ECM (myocardium and pericardium), secondary sources such as small intestine submucosa, omentum, placenta, liver, lung, skin, and urinary bladder matrix have also been studied as potential dECM sources for cardiac tissue engineering applications.^61,85,123–129^ Although primary sources offer cardiac-specific biochemical cues and structural composition, facile process, ease of obtaining, and improved reproducibility are the main reasons behind using secondary dECM sources for cardiac regeneration.^130^ Several studies have been conducted to compare the characteristics of various dECM sources with primary cardiac dECM. Hong et al. found that murine cardiac and skeletal dECM possessed similar fiber structures.^126^ Higuchi et al. showed that murine liver dECM had lower protein concentration compared to the murine cardiac dECM and cardiac dECM can lead to higher cardiac differentiation of seeded endothelial stem cells (ESCs) than the liver dECM.^125^ Merna et al. decellularized porcine lung and cardiac ECM and demonstrated that lung dECM distinctively expressed collagen II and collagen IX while cardiac ECM distinctively expressed collagen VII, fibrinogen, and heparin sulfate.^127^

Based on the reported studies, it can be concluded that cardiac ECM composition has an important role in the fate of incorporated stem cells and ECM remodeling. Further clinical studies and investigations are yet required to determine which dECM source results in the most effective therapy.

### Modulation and characterization

#### Mechanical and rheological properties

As discussed in the previous sections, injection of cell-laden or cell-free hydrogels into the heart wall is one of the promising strategies for improving heart function following myocardial infarction.^90^ dECM-based injectable hydrogels are among the top-listed options, due to their intrinsic properties such as high protein and polysaccharide content which supports different cell activities and functions such as migration, proliferation, and differentiation. Despite these advantages, the main drawback when it comes to the dECM hydrogel injection is its inadequate mechanical strength. It is known that stiffness in the range of 20 to 50 kPa is required for the stabilization of the heart tissue.^131^ Different methods of fine-tuning the mechanical properties of dECM hydrogels to match with that of the native myocardium are the scope of the following section.

##### Cross-linking

Chemical crosslinking is one of the main strategies applied to improve the stiffness of dECM hydrogels. In a study by Singelyn et al.,^132^ a glutaraldehyde (GA) agent was employed for modifying the mechanical properties of the myocardial hydrogel. Results suggest that although using 0.1% GA as a crosslinker increases the stiffness of myocardial hydrogel from 5 Pa to 136 Pa and prolongs the degradation time, further increase of GA concentration may be problematic due to cytotoxicity and calcification. Furthermore, damaging elastin in GA-treated heart valves threatens the elasticity.^133^ the increased stiffness of GA-treated tissues is known to be due to the presence of only rigid carbon-carbon bonds in the GA backbone structure.^134^

Williams et al. showed that transglutaminase (TG) crosslinker can enhance the tensile mechanical characteristics of Fibrin-cardiac ECM hybrid hydrogel.^135^ The results showed that adding 12 and 120 µg/ml TG, increases the elastic moduli of hydrogel to 13.7 ± 4.8 kPa and 32 ± 4.6 kPa, respectively. Assessment of TG on hybrid hydrogel of fibrin and both neonatal and adult ECM revealed that the age of ECM did not influence the network formation. However, the final stiffness of hybrid hydrogel crosslinked with 1.2 µg/ml GT led to higher network maturity.

It is proved that a stiffness range of 100–300 Pa induces the vascular differentiation of MSC cells.^136^ Genipin as a natural crosslinker agent extracted from Gardenia Jasminoides Ellis fruit was exploited to enhance the mechanical characteristics of cardiac matrix hydrogel (stiffness of 200–300 Pa) and induce endothelial differentiation of MSCs. Although genipin crosslinking was found to down-regulate the expression of early endothelial cell markers such as CD31 and VE-cadherin, it enhances the expression of mature endothelial cell markers of vWF.^137^ The results of this work demonstrated that the addition of 1–2 mM genipin to cardiac-derived hydrogel does not cause negative effects on cell viability. Compared to GA, genipin exhibits less cytotoxicity which introduces it as a superior biocompatible crosslinking agent.^138^

The preservation of elastin plays an essential role in the mechanical properties of decellularized tissue products as a resilient and cyclic load-bearing component.^139^ Jin et al. have evaluated the glycidal methacrylated porcine pericardium (GMA-PP) cross-linked with ammonium persulfate (APS) and sodium hydrogen sulfite (SHS) as a radical polymerization initiator.^133^ Increasing GMA concentration from 1% to 5% resulted in fewer free available amine groups on PP ECM as a result of the reaction of the amine and epoxy groups that prolongs the biodegradation rate by stabilizing the collagen component of ECM. Furthermore, results prove that GMA-treated tissues lose lower mass in presence of elastase, indicating that radical polymerization crosslinking can protect elastin more effectively than other modification systems such as GA-treated tissues.^133^ Furthermore, it is known that moderate stiffness is required for heart valves to provide a quick response to blood flow during the opening and closing stages to decrease heart burden.^140^ Results of this work suggest that 5% GMA-PP provides lower secant modulus and higher extensibility compared to GA-PP which introduces 5% GMA-PP as a softer and more ductile tissue.^133^

**Figure 5.**
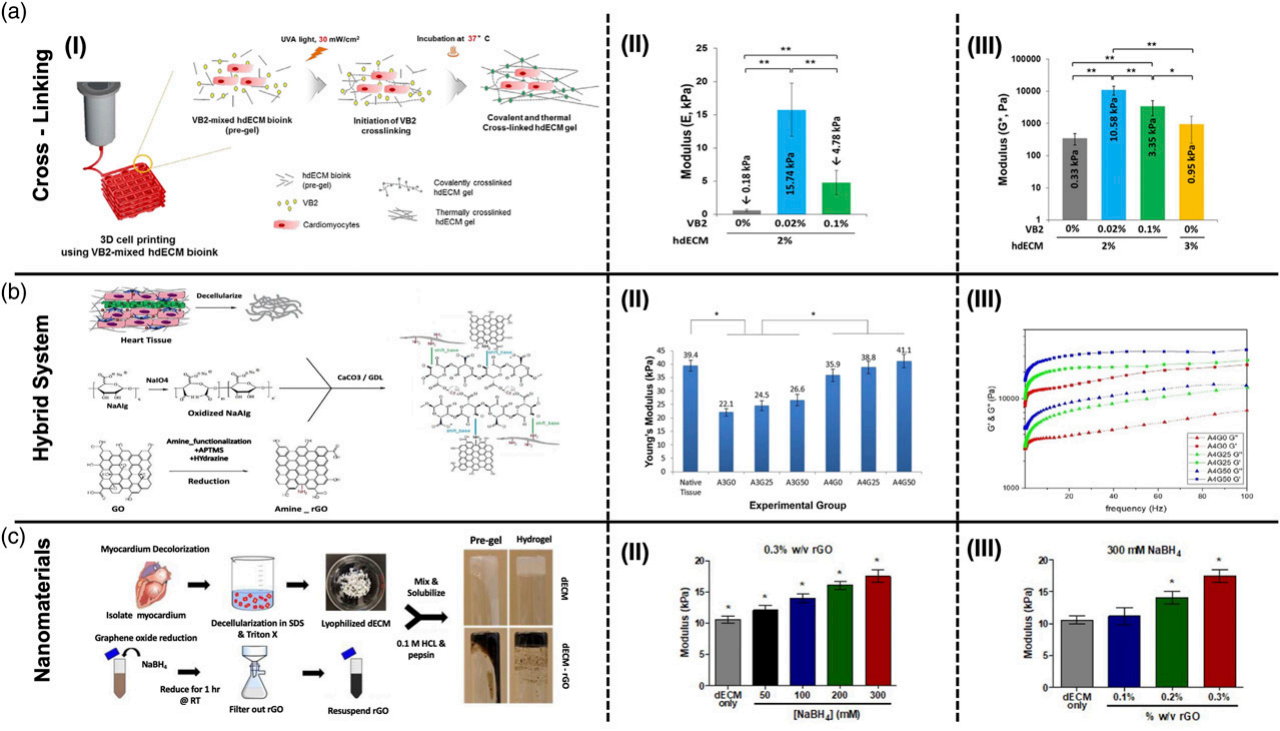
Examples for three main categories of tailoring mechanical properties of cECM derived hydrogels: (a) Crosslinking method (i) Schematic illustration of photocrosslinking using VB2, (II) Compressive modulus of bioinks containing different concentrations of VB2 at 20% strain (III) Dynamic complex modulus of bioinks containing different concentration of VB2 at 1 rad/s^141^; (b) Hybrid systems (i) Schematic overview of possible chemical interactions between APTMS-functionalized rGO with hybrid OA-cECM matrix, (II) Tensile Young’s modulus of hybrid hydrogels containing various concentration of OA and rGO, (III) Dynamic moduli of hybrid hydrogels containing various concentration of rGO^93^; (c) Nanomaterials method (I) Synthesis procedure of cECM hydrogel containing rGO, (II) Compressive modulus of cECM hydrogel containing 0.3 %w/v rGO reduced with different molarity of NaBH_4_ agent, (III) Compressive modulus of cECM hydrogel containing different concentration of rGO reduced with 300 mM NaBH_4_ agent.^143^

Recently, vitamin B2 (VB2) also named riboflavin has gained tremendous enthusiasm as a photosensitizing agent for improving the mechanical properties and printability of dECM bioinks. Irradiation of VB2 by visible or UVA light excites an electron to a higher energy state, which contributes to the generation of reactive oxygen species (ROS) through the reaction of intersystem crossed triplet VB2 with atmospheric triplet oxygen. Subsequently, the reaction between the generated ROSs and various ECM molecules such as collagen yields covalent crosslinking.^141,142^ Schematic illustration of photocrosslinking process using VB2 is shown in Figure 5(A-I). Photo crosslinking of a bioink based on decellularized cardiac hydrogels by using VB2, resulted in a compressive modulus of 15 kPa (Figure 5(A-II)), which is similar to the stiffness of native cardiac muscle tissue. However, both compressive and dynamic modulus of the bioink deteriorates when increasing the VB2 concentration from 0.02% to 0.1% (Figure 5(A-II) and (A-III)) and slight cytotoxic effects are observed.^141,142^

##### dECM-based hybrid systems

Blending dECM-based materials with other natural or synthetic biopolymers is a reliable and popular strategy for the treatment of heart failure. In this regard, different polymeric combinations have been reported. For example, the hybrid high G block alginate-dECM hydrogel presented over a 300-fold rise in storage modulus when compared to the dECM hydrogels. Furthermore, it has been shown that the addition of dECM to alginate hydrogel improves their compressive modulus.^90^ Musavi and coworkers combined bovine cardiac dECM with oxidized alginate, amine-functionalized rGO, and CaCO_3_/glucono-δ-lactone (GDL) cross-linking agent to fabricate an electroconductive hybrid hydrogel (Figure 5(B-I)). The Schiff's base reaction between aldehyde groups of OA and amine groups of the dECM was utilized to improve the mechanical properties of the hydrogel. As shown in Figure 5(B-II), rising the concentration of OA from 3 to 4 %w/v enhances the tensile modulus of OA-dECM hydrogel . They also demonstrated that the incorporation of amine-functionalized rGO into OA-dECM hydrogel, can increase the tensile modulus to around 41 kPa (Figure 5(B-II)). Furthermore, storage and loss modulus values can be modulated by altering the concentration of the nanosheets in the hydrogel (Figure 5(B-III)).^93^ This value is close enough to the mechanical characteristics of the native myocardium and suggests that injectable amine-rGO incorporated OA-dECM hydrogel can tolerate contacting forces in the infarct area.

In another study, the incorporation of type I collagen into myocardial hydrogel was also shown to increase the storage modulus. A hydrogel system consisting of 75% decellularized myocardial hydrogel and 25% collagen type I with a storage modulus of 8.6 Pa was found to be an appropriate matrix for cardiac differentiation of human ESCs. Signals generated from their naturally-occurring bioactive molecules and growth factors support and induce the differentiation of ESCs. Furthermore, the more dECM component, the better the beating amplitude of the encapsulated cells.^144^

The softness and instability of porcine cardiac-derived ECM (pcECM) hydrogel hampers its application for cell culture, mainly because it cannot withstand the forces applied during the contraction and relaxation of the heart. The addition of chitosan as a strengthening biomaterial to a genipin crosslinked pcECM hydrogel increases the storage modulus by around ∼6 folds. Advantageously, the incorporation of chitosan results in a dense network with thinner fibers and larger pore sizes which leads to more collagen secretion by MSC stem cells.^92^

Silk fibroin is another natural biopolymer that has been used for tailoring the mechanical properties of cardiac-derived ECM (cECM) hydrogel.^91^ It has been shown that the addition of 2 to 4 %w/v of silk fibroin in hybrid hydrogels yields higher stiffness.^91^ The main limitation of silk fibroin is the lack of cell binding sites and therefore requires chemical modification to improve this property. The incorporation of cECM into silk fibroin hydrogel has been shown to improve cell functions *in vitro*, due to the presence of adhesive peptides and promote ECM remodeling and vascularization *in vivo*^62,91^ In another work by Stoppel et al., the effect of cECM addition to silk hydrogel was investigated.^62^ Results supported that by incorporating the cECM, scaffolds with mechanical characteristics analogous to the native heart (25–50 kPa) can be acquired, and increasing the concentration of cECM further improves the bulk elastic modulus.

dECM-polyethylene glycol hybrid system was also investigated as an ideal candidate to improve the stiffness owing to the biocompatibility, non-immunogenicity, and inertness of PEG. PEG can be incorporated into a nanofibrous network of dECM materials using either the direct reaction of carboxylic acid-functionalized PEG (PEG-NHS) with amine residues on the proteins, or the addition of multi-armed PEG acrylates into the acryl-functionalized proteins. The reaction of PEG acrylate with itself, as well as acryl-functionalized polymers, results in a stiffer structure than the PEG-NHS. Radical photo-polymerization of the myocardial matrix with multi-armed PEG acrylate increases the storage modulus of hybrid hydrogel without inhibiting cell attachment or cell migration within the hydrogel.^91,145^ Even though easy cell migration through PEG-NHS hybrid hydrogels limits the *in vitro* 3D culture applications, its injectability and slow biodegradation rate are of benefit for *in vivo* studies. Photo-crosslinked PEG hybrid hydrogels, on the other hand, are considered an appropriate platform for *in vitro* cell encapsulation.^146^

Along the same vein, one of the major drawbacks of xenogeneic decellularized heart valves is the activation of platelets in direct contact with collagen. It has been shown that both the cellular and plasmatic clotting systems can be deactivated by polymer coating of the decellularized heart valves.^147^ However, the results of the study by Stamm and colleagues illustrate that coating the decellularized valve tissues with poly (3-hydroxybutyrate) and poly (4-hydroxybutyrate) improves the mechanical characteristics of decellularized tissue including suture retention strength and tensile strength and helps to improve the remained antigenicity.^147^ Despite the biocompatibility and mechanical properties of decellularized valve tissue caused by poly (3-hydroxybutyrate) treatment, coating the xenogeneic valve with poly (4-hydroxybutyrate) results in softer tissue with earlier calcification *in vivo*.

##### Nono-reinforced dECM hydrogels

In the last decade, nanomaterials have gained great attention in the field of TE by enhancing mechanical, rheological, and electrical properties. However, this modification method may be limited to specific content of the nanoparticles that do not cause cytotoxic effects.^143,148^ Another concern about using nanomaterials is their uniform dispersion into the polymeric matrixes which in some cases can not be obtained without a further surface functionalization process.^149^ Here in the following section, the different types of nanomaterials applied to improve the properties of cdECM hydrogels will be discussed in detail.

The addition of reduced graphene oxide nanoscale flakes to the decellularized ECM hydrogel helps to improve the mechanical properties to fit the range of modulus for cardiac tissue engineering. Tsui et al. combined decellularized cECM with rGO to produce an electroconductive cECM-rGO hydrogel (Figure 5(C-I)). Results demonstrated that the degree of reduction (Figure 5(C-II)), as well as rGO content (Figure 5(C-III)) in the hydrogel, plays the most significant role in modulating the mechanical characteristics of the composite dECM hydrogel.^143^ The higher degree of reduction leads to an sp^2^ hybridized graphene lattice structure, which causes higher electrical conductivity and mechanical strength. Furthermore, residual oxygen-containing functional groups in rGO, support the adsorption of proteins and other biomolecules that play role in cellular activities.^143^

As another generation of carbon-based nanomaterials, the incorporation of carbodihydrazide functionalized multi-wall carbon nanotubes into the pericardial hydrogel could enhance the stiffness of hydrogels and increase the storage modulus by 67%. However, the obtained modulus is still far below the modulus of the native myocardium (20–25 kPa).^149,150^

Recently, laponite nano-silicates have been used to enhance the printability of a hybrid cdECM-derived and PEGDA bioink. It was shown that the incorporation of laponite nanoparticles could improve the shear-thinning property and printability of the polymeric bioink.^151^ Laponite is a disc shape nanoparticle that bears a negative charge on the surface and a positive charge on the edges at pH values lower than 9.^152,153^ The formation of laponite-protein electrostatic interactions in laponite containing cardiac dECM-based bioink causes a solid-like characteristic to bioink and enhances its stiffness. In addition to the effect of laponite nano-silicates, it was shown that different concentrations of PEGDA as a crosslinker can modulate the mechanical properties of cardiac dECM from 5 to 15 kPa in healthy tissue to 30–100 kPa in diseased and fibrotic heart tissues to fabricate 3D bioprinted models for tissue modeling or drug screening applications.^151^

#### Electrical properties

Synchronous cardiac contraction via electrical activation is an essential factor affecting myocardial function.^154^ To provide a contractile and functional cardiac patch, bioreactors play a crucial role in electrical signal delivery. Overall, electrical stimulation of cardiac patches helps better cell coupling, induces cell elongation, and improves the amplitude of synchronous contraction. Moreover, the design of such bioreactors should facilitate oxygen penetration to the whole tissue-engineered organs.^154^

The electrical stimulation of decellularized rat hearts, recellularized with rat aortic endothelial and neonatal cardiac cells has led to the formation of contractile cardiac tissues, which is a pioneer research work in the field of preparing functional decellularized organs.^14^ 8 days after recellularization, the cultured heart tissue exhibited contractile behavior in response to electrical stimulation, which led to rising in left ventricular pressure and repolarization. To investigate the possibility of using injectable hydrogels derived from decellularized tissues for arrhythmogenesis, the arrhythmias induction was examined by electrical stimulation of rats treated with a decellularized myocardial hydrogel. The results showed no increase in the occurrence of arrhythmia via injection of myocardial hydrogel into the infarct area.^83^

Electrical stimulation serves as a method for engineering cardiac muscle with aligned connected cells. Applying *in vitro* direct electrical field has been proven to influence the migration and elongation of cardiac fat-derived stem cells. Results show that the cells align in a perpendicular direction to the applied electric field. Electrical stimulation also disassembles gap junctions, decreases the gap junction number per cell nucleus, and upregulates the gene and growth factor expression.^155^

The decellularized cardiac tissues are also considered for improving cardiac function post-MI by preventing scar formation and controlling the wall stresses. However, the matured cardiomyocyte cells in the scaffold are not independently electroactive and need electrical stimulation for efficient cardiac function.^82^ Therefore, the induction of electro-conductivity of the dECM-based scaffolds is an essential factor, which initializes the propagation of electrical signals during the early stages of cell and tissue maturation, most importantly before the development of gap junctions.^143^ To this end, various nanoscale conductive materials have been incorporated into the scaffolds as an electrical coupler between cells, which will be discussed in the following section.

##### Carbon-based nanomaterials

The presence of reduced graphene oxide)rGO (flakes in dECM hydrogels improves the electro-conductivity of the scaffold. Deposited rGO nanoflakes on the walls of the porous decellularized myocardial hydrogel, were reported to provide an appropriate way for cells to bridge electrically with one another.^151^ The addition of 3-(2 aminoethyl amino) propyltrimethoxysilane (APTMS)-functionalized rGO to hybrid oxidized alginate-myocardial dECM hydrogel was demonstrated to improve electroconductivity and provide a suitable scaffold for cardiac tissue engineering.^93^

The inherent hydrophobicity of CNTs causes aggregation and poor dispersion in water. To address this issue, the functionalization of these nanostructures has been widely investigated by researchers. For instance, the incorporation of carbodihydrazide (CDH)-functionalized MWCNTs (CDH-MWCNTs) into the decellularized pericardial gel increases the electrical conductivity and induces the synchronous beating of cardiomyocyte cells, as reported by Roshanbinfar et al.^149^ The higher expression of Cx43 gap junction protein in the CDH-MWCNT containing pericardial gel, compared with the pure pericardial gel, demonstrates better cell-cell communications and subsequently promotion of intercellular electrical coupling and contractile behavior.^149^ In another study by this research group, the interaction between positively charged CDH-CNTs and negative cardiomyocyte cell membranes was shown to improve their functions. The high aspect ratio of CNTs as a nanofiller in the hydrogel forms the conduction anchor points which self-assemble as a conductive pathway. The enhanced ionic transportation through these pathways leads to the self-organization of cardiomyocytes as an aligned cellular structure with a unidirectional orientation. This helps better electrical signal propagation between coupled cells as well as improved beating.^150,156^

##### Metal-based nanomaterials

The deposition of a thin layer of Au nanoparticles on the decellularized omentum tissue has been reported to have attenuation effects on fibroblast proliferation, leading to the preservation of the initial fibroblast to cardiomyocyte ratio. Controlling the ratio between contracting and non-contracting cells causes a stronger contraction force in the cardiac patch. Au nanoparticle deposition decreases the threshold excitation voltage required for synchronous contraction of the patch due to the improved coupling between the cells.^94^ Myocardial ECM-derived hydrogels loaded with gold and laponite nanoparticles were also demonstrated to improve the expression of cardiac-specific markers, due to the improved electroactivity and bioactivity.^157^

##### Conductive polymeric materials

Polypyrrole (Ppy) is known as a biocompatible conductive polymer with a low inflammatory response, which suffers from poor mechanical properties. Ppy as a polycationic polymer has the potential to serve as an appropriate substrate for attracting cell membranes with a negative charge and therefore, enhancing cell attachment.^158^ Using Ppy in combination with decellularized cardiac ECM hydrogel was studied by Parchehbaf et al.^158^ They observed a 30-fold increase in conductivity and improved expression of Cx43 in their dECM-Ppy scaffolds, which indicates better electrical signal propagation and force generation during the contraction of cardiomyocyte cells.^158^

#### Immunomodulation

Cardiac ECM is a complex structure that plays a prominent role in cell regulations. Not only it contains various proteins, polysaccharides, and glycoproteins for cell attachments and proliferation but it also includes bioactive motifs promoting important cellular activities such as modulating immune responses.^159^ Immunomodulation should be precisely investigated in designing new biomaterials for tissue engineering. It encompasses all therapeutic interventions aiming to make scaffolds effectively integrate with host cells, and prevent infection in terms of immunodeficiency.^160^ Briefly, recognition of implanted materials in the body would induce inflammatory responses stemming from neutrophils and macrophages followed by inflammatory cytokine secreted from dendritic cells, B cells, and T cells. Therefore, newly designed tissue constructs should alleviate the host immune responses.^161^ It has been reported that dECM contains immunomodulatory sites binding to immune cell receptors for regulating inflammatory responses. Dziki et al. have shown that bioactive scaffolds could impact macrophage polarization from a pro-inflammatory M1 phenotype to a pro-reparative M2 phenotype.^162^ However, tissue source, processing method, age, and antigen removal procedure could directly affect the dECM composition and immunomodulatory properties.^163^

To cite an example, Meng and his coworkers have reported in their studies conducted on primary rat macrophages that urinary bladder dECM induced more prostaglandin E but less arginase TNFα and nitric oxide in comparison with the brain-derived ECM. In another study, Loprestine et al. have shown that the small intestine submucosa dECM of a 52-weeks pig could not promote the inflammatory iNOS as much as a 12-weeks pig. Accordingly, it is difficult to control the composition of xenogeneic and allogenic decellularized tissue. Hence, dECM should be functionalized with specific peptides to make its composition and immunomodulatory properties more tunable.^164^

##### Arginylglycylaspartic acid

Arg-Gly-Asp (RGD), a peptide motif responsible for pairing cellular integrin to ECM, is the amino acid sequence that exists on ECM proteins such as collagen, fibrinogen, laminin, and vitronectin. Recently, it has been reported that the RGD domain can display numerous immunomodulatory impacts on immune cells. In the case of dECM soluble hydrogels, RGD peptides can be hybridized with the hydrogels to evoke anti-inflammatory responses.^163^ Zeveri et al. have studied the role of RGD on macrophage activation. They realized how effectively RGD peptides could decrease macrophage phagocytosis and hamper cytokines, TNFα, and IL-6, secretion.^165^ To cite another example, Bryant et al. have functionalized PEG prepolymer with RGD domain and studied the effect of this polypeptide on cell adhesions and promoting anti-inflammatory response. They reported that PEG-RGD hydrogels represented longer cell viability and higher inflammatory cell layer thickness compared to PEG-only hydrogels. Thus, the addition of RGD peptides to dECM hydrogels would positively affect its immunomodulation responses.^166^

##### Matrix metalloproteinase

Matrix metalloproteinase (MMP) also known as matrixins are calcium-dependent zinc-containing endopeptidases that are capable of degrading an extensive range of ECM proteins as well as processing various bioactive molecules.^167^ MMPs play essential roles in cell migration, attachment, proliferation, differentiation, angiogenesis, apoptosis, and host defense.^168^ In fact, they cleave cell surface receptors and release the apoptotic ligands to get involved in the cytokine/chemokine inactivation process.^169,170^

In the context of immune systems, MMP-sensitive peptides can be incorporated into implanted materials to improve their integration with native tissues and to regulate the level of immune cell invasion. Fonseca et al. have investigated the immunogenicity of the ECM hydrogels treated with MMP-sensitive peptides. The effects of MMP-sensitive peptides on the activation of human monocyte-derived immature dendritic cells were assessed in their study. They have shown that activation of biomarkers including CD83 and CD86 are less expressed in hydrogels treated with MMP peptides compared to the control group (untreated hydrogels). Therefore, these peptides stop the up-regulation of dendritic cell activation biomarkers.^171^ In another study done by Amer et al. PEG prepolymers were crosslinked with MMP-sensitive peptides to form hydrogels. They assessed the effect of macrophage cell line implantation on hydrogel’s degradation rate. It was shown that samples did not experience significant degradation when they were incubated in macrophage-conditioned media. Their observations were in accordance with the fact that macrophages might secret tissue inhibitors of metalloproteinases to prevent the function of MMPs and to make PEG hydrogels maintain their stability. As a result, it has been proved that MMP-sensitive peptides have crucial impacts on immune cell modulations.^172^

#### Dynamic remodeling

It has been reported that cardiac ECM remodels when myocardial infarction occurs. The composition of infarct ECM alters and its stiffness increases several weeks after MI during the later stages of remodeling.^173^ The remodeled decellularized matrix influences the cellular phenotypes remarkably. In this case, assessing the dECM composition with time following infarction is crucial in fabricating ideal scaffolds to maximize spontaneous tissue repair and minimize the detrimental outcomes of heart failure,^174^ In the following paragraph, the dynamic remodeling of several ECM components during MI will be further elucidated.

Infarct dECM collagen assessments have revealed collagen organization and deposition into fiber bundles increase dramatically over time.^173^ Further analysis confirmed that infarct ECM contained more diverse matrix proteins including collagen I, laminin, collagen IV, and collagen XV, 1 week after MI compared to the composition of the infarct 4 weeks post-MI.^174^ Phatharajaree et al. have related reductions in ECM protein contents to MMP expression increment 1 week after coronary artery ligation.^175^ Other work has shown a rapid rise in periostin, fibronectin, and collagen XI, 7 days after the artery ligation. In contrast to collagen XI which adversely influences cardiac function after MI, fibronectin contributes to cardiac wound healing. These observations suggest that the new cardiac tissue will be remodeled negatively due to the elastin and collagen V shortage. Furthermore, the remodeled ECM could not function normally as its main protein, collagen, is not sufficiently present.^176–179^ Therefore, the infarct dECM represents important changes in both mechanical and structural characteristics with remodeling after infarction.

ECM stiffness and composition are important factors in dynamic remodeling as they directly affect cellular responses. These alternations should be applied to the newly designed scaffold to understand how the stiffness and composition variation would stimulate cell differentiation toward a cardiac lineage. It is expected that the new microenvironment dynamically up-regulates the potential cardiac differentiation.^174^ Consequently, the characterization of the disease matrix components and cell fate as a function of myocardial remodeling is crucial for scientists. They can exploit appropriate techniques for cell implantations to enhance tissue regeneration and alleviate the severity of heart diseases such as MI.

#### Bioactivity

Bioactive materials used as structural scaffolds have important signals and cues to support appropriate cell health, function, and tissue repair.^180^ Simple bioactive scaffolds are comprised of ECM proteins like collagen type I. More complex bioactive scaffolds are obtained through the decellularization of native tissue. This ultra-structural composition contains matricellular proteins and growth factor reservoirs.^181,182^ Svystonyuk et al. have investigated epicardially implanted acellular bioactive scaffolds for ischemic injury. They treated the bioactive scaffolds with exogenous growth factors to emphasize their capability as a platform therapy. Also, they studied the infarct area replaced with the bioactive scaffold and confirmed the arteriole formation next to the surviving cardiomyocyte islands. It was proved these beneficial results were because of bioactive components existing inside the scaffold and were not the effect of myocardial restraint influences,^180^ Thus, bioactive scaffolds implanted in the body could provide a new signaling circumstance in the heart which could accelerate endogenous cardiac regeneration.

Degraded ECM components leverage bioactive scaffold constructs including structural molecules like laminin, fibronectin, collagen, glycosaminoglycan, and matrix-bound nanovesicles (MBV) resembling exosomes in size and structure. It has been shown through *in vitro* cell culture that MBV can recapitulate phenotypical and functional effects associated with ECM bioscaffolds.^183^ Table 2 presents a list of bioactive ECM constituents that play significant roles in constructive tissue remodeling.

**Table 2.** Bioactive ECM components that play important roles in constructive tissue remodeling.

ECM components	Examples	Function	References
Structural proteins	Collagen, laminin, fibronectin	Provide tensile strength to tissues	^184^
Glycosaminoglycan	Heparin, heparin sulfate, hyaluronic acid, chondroitin sulfate	Modulation of enzyme activity, regulation of cell growth	^185^
Growth factors	VEGF, TGFβ, bFGF	Promote angiogenesis, mitogenesis, and cellular differentiation	^186^
MBV	MBV contains microRNA, protein, and lipid cargo	Induce macrophage polarization and stem cell differentiation	^187^
Matricryptic peptides	Carboxy-terminal telopeptide region of the collagen IIIα molecule	Chemoattractant for progenitor cells.	^188^

The combination of bioactive scaffolds with other tissue engineering approaches would act as a key to speeding up endogenous cardiac regeneration. Additionally, bioactive scaffolds used as standalone therapy could be exploited for different purposes. Not only they could deliver therapeutic agents directly to the heart but they could also be coupled with cell therapy techniques to resolve cell engraftments related to intracoronary and intramuscular delivery.^183^ In conclusion, bioactive scaffolds are tunable platforms that could be engineered toward the specific clinical characteristic of the patients to provide more personalized and precise therapy.

### Fabrication methods

The native cardiac tissue is a complex organ and consists of various cell types, different ECM components and biomolecules, and biophysical and biochemical cues. To engineer cardiac tissue, the ideal scaffold should emulate the architectures, physiochemical properties, and anisotropic mechanical and electrical features of the native tissue. The native ECM provides geometrical or biochemical cues and promotes cardiomyocyte self-assembly which generates synchronous contraction.^189,190^ To mimic the native cardiac tissue, the designed scaffold should possess several essential features:

i. Interconnected porous structure with various pore sizes to facilitate cell homing and migration, vascularization, and nutrient and oxygen diffusion,
ii. Nanoscale components which are abundant in native ECM and play a critical role in regulating cellular behavior such as attachment and maturation,
iii. Regulating the numbering and positioning of different cell types in specific locations in cardiac constructs.

However, there are still many challenges that need to be addressed before these engineered platforms can unleash their promise in clinical studies. Some of the most important features of the cardiac tissue which are challenging to mimic include the recapitulation of complex architecture, aligned fibers embedded into a 3D honeycomb-like structure, and the inclusion of a variety of cell types such as cardiomyocytes, cardiac fibroblasts, vascular smooth muscle cells and endothelial cells of myocardial tissue.^40,191^ Another key challenge, which remains unmet in the traditional tissue engineering approach is promoting the vascularization in cardiac constructs with a thickness of more than 100–200 μm.^192,193^ The implementation of pre-vascularization strategies is one way to enhance the efficient blood supply to the tissues. To this end, KC et al. examined several critical factors involved in the interaction among decellularized porcine myocardium slices (dPMS) and reseeded human mesenchymal stem cells (hMSCs) and rat adipose tissue-derived stem cells (rASCs) to fabricate pre-vascularized heart tissues. Results indicated that dPMS thickness affected cell seeding efficiency and proliferation. After 1 day of cell culture, dPMS induced angiogenesis and endothelial differentiation of hMSCs. A similar effect was observed in rASCs after 5 days of culture. When applying full-thickness decellularized tissue patches, complications such as poor cell survival and uneven distribution of cells arise due to the limited available blood supply, which could be resolved by utilizing a proper vascularization approach.^194^

Extensive research efforts have been put into the fabrication of biomimicry cardiac scaffolds via different methods and technologies including electrospinning, 3D bioprinting, and lithography.^130,195–197^ In the past few decades, several research works have shown that the manufacturing process can highly affect the structural and functional characteristics of scaffolds and cellular behavior. ^92,119,196,198^ The ongoing progression of these fabrication techniques has opened up new avenues in the pursuit of functional cardiac tissues, however, there are several advantages and disadvantages associated with each of them. In the following part of the review, the current approaches used for the fabrication of heart-derived dECM-based cardiac tissue construct will be discussed.

#### Bioprinting

3D bioprinting is a powerful tool for fabricating tissue-analogous constructs to study cell behavior for *in vitro* disease modeling, drug screening, and tissue engineering. 3D bioprinting has several advantages including better control over the test bed size, shape, architecture, and material constituents compared to traditional biofabrication techniques, and facilitates the homogenous distribution of cell populations.^26^

The key factor of 3D bioprinting approach is the selection of an appropriate bioink that can emulate the physicochemical cues of the tissue-specific microenvironment. For extrusion bioprinting, the bioink should possess the following properties: (i) relatively high viscosity, (ii) strong shear-thinning behavior, and (iii) post-printing rapid crosslinking.^199,200^

Cardiac dECM is composed of various cardiac ECM biomolecules and components that are essential for regulating cardiomyocyte function, and therefore can serve as a biomimicry and bioactive bioink.^201^ Therefore, several studies have exploited dECM bioink for 3D bioprinting of cardiovascular constructs and showed that improved cardiac function can be acquired through enhanced cell adhesion, differentiation, and maturation.^130,202^ In support of this concept, Das et al.^195^ developed two types of hydrogel bioinks for the development of 3D-printed cardiac tissue models. They assessed the effect of microenvironment factors such as bioink component and culture condition (static vs dynamic culture) on cardiomyocyte behavior. Results have shown that using dECM, even at low concentrations, could enhance cardiomyocyte maturation and promote cell differentiation. Furthermore, these results highlight that the presence of dECM can affect the viscoelasticity of the microenvironment thereby promoting cell-cell and cell-matrix interactions.^195^ In addition, Pati et al.^203^ reported that the dECM-based construct accelerates the therapeutic effect of the 3D printed scaffolds for cardiac repair and observed higher expression of cardiac-specific genes such as Myh6, β-MHC, and Actn1 compared with collagen-based constructs under similar culture condition. 3D bioprinting is capable of positioning one or more polymers or hydrogels or both at precise locations. In this regard, combining 3D bioprinting and dECM is a powerful approach to produce cardiac patches.

Although the dECM bioink shows great biocompatibility, it cannot maintain consistent printability over long processing times, because the main component of the dECM bioink –collagen—is thermo-sensitive and its rheological property changes during the printing process.^204^ Another challenge in printing dECM bioinks is their weak mechanical properties, low viscosity, and slow crosslinking speed which result in poor printability and low structural fidelity.^201,205^ Therefore, several studies have focused on the combination of other biomaterials and the incorporation of nanostructure, or using two-step crosslinking approaches to improve the viscosity and printability of dECM-based bioinks. Additionally, the methacrylation of dECM-based bioinks or the addition of photo-crosslinkers can be another useful strategy to improve their printability.^143,199,206^ To cite an example, Kim et al. introduced a novel bioink including dECM micro-particles loaded into a gelatin mixture.^207^ They reported dECM micro-particle instead of the solubilized form of dECM with improved 3D printability and mechanical characteristics. In another study, Bejleri et al. used a combination of cardiac dECM with GelMA to enhance its printability via thermo/photo dual-crosslinking.^202^ It was shown that the presence of dECM in the bioprinted cardiac patch (GelMA-dECM) improves the differentiation and angiogenesis of human cardiac progenitor cells by the release of paracrine factors, compared to pure GelMA patch. Similarly, Jang et al. developed a pig heart dECM bioink combined with human cardiac progenitor cells in which the physical and rheological properties were improved using vitamin B2 and UV light.^130^

**Figure 6.**
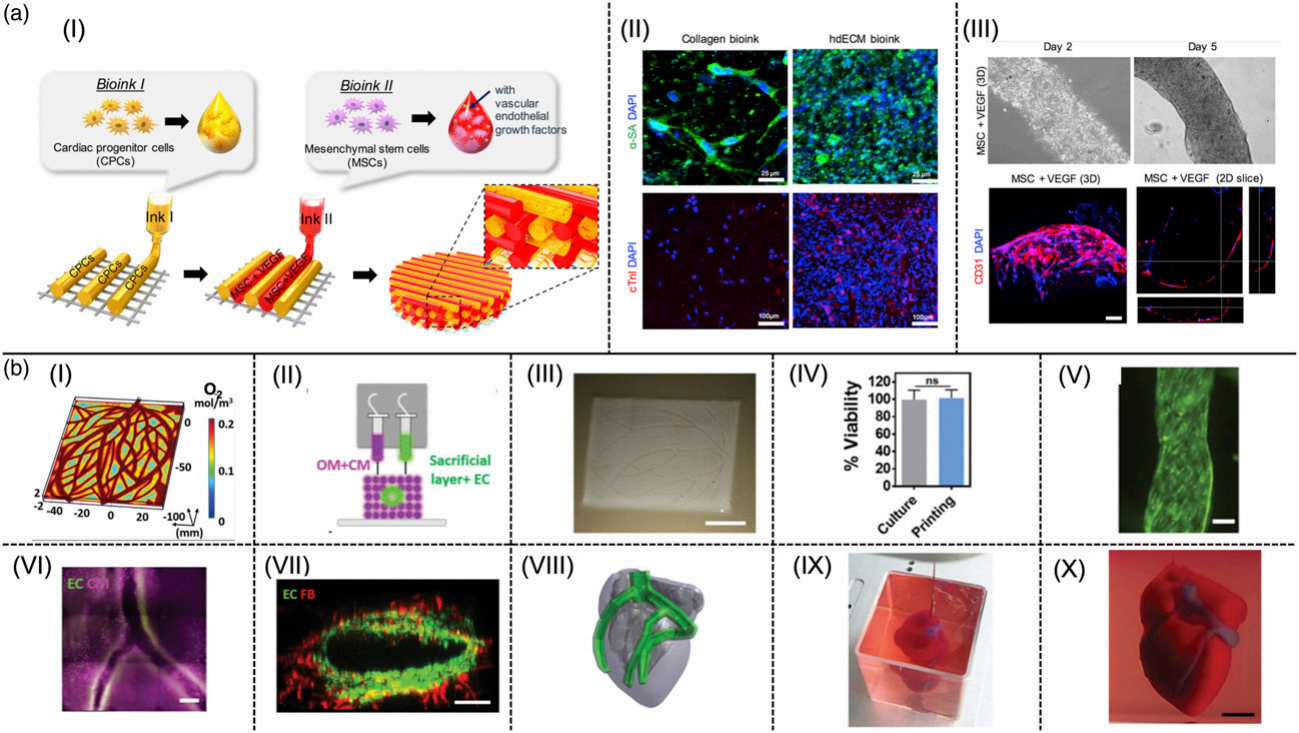
Multicellular components pre-vascularized patch, (a) (I) Schematics of the preparation via 3D bioprinting, (II) Immunofluorescence staining against α-sarcomeric actin (green) and cardiac troponin I (red) after 1 week, (III) Microscopic imaging of MSC+VEGF lines after 2 and 5 days of culture (magnification 200x) and immunofluorescence staining against CD31 (red) after 5 days (Scale bar: 50 μm)^209^; (b) 3D printed personalized cardiac patch, (I) The designed model with sufficient oxygen diffusion, (II) Schematics of the printing process and the different cellular bioinks, (III) A bioprinted vascularized cardiac patch, (IV) Post-printing cell viability, (V) A bioprinted cellular lumen, constantly layered with GFP-expressing ECs, (VI) A bioprinted iPSCs-derived cardiac patch where the cellular lumens (CD31 in green) are observed within the cardiac tissue (actinin in pink), (VII) Cross-sections of a blood vessel, indicating the interactions of GFP-expressing ECs and RFP-expressing FBs (Scale bar: 100 μm), (VII) The CAD model of the human heart, (IX, X) A bioprinted heart inside a support media (Scale bar: 0.5 cm).^211^

3D bioprinting can also pave the road toward the development of vascularized engineered cardiac constructs.^208^ To this end, various strategies have been employed such as the incorporation of multiple cells or biofactors in the scaffolds,^142,209^ designing perfusable microchannels,^210^ and the creation of vascular networks.^211^ Park and coworkers showed a concomitant technique to evaluate the synergistic effects of two types of stem cells to induce vascularization and aid cardiac regeneration through the constant delivery of paracrine factors.^142^ They injected hiPSC-CMs intramyocardially and implanted a dECM-based 3D bioprinted cardiac patch loaded with human mesenchymal stem cells epicardially. Through this approach, they developed a strategy for cardiac repair that can concurrently rejuvenate both the myocardium and vasculatures using two types of stem cells.^142^ Jang and coworkers developed a pre-vascularized patch with multicellular components including cardiac progenitor/mesenchymal stem cells via 3D bioprinting.^209^ To fabricate this patch, first, 2 layers of PCL were printed as mechanical support, then bioink I (porcine heart dECM with CPCs) and bioink II (porcine heart dECM with MSCs +VEGF) were alternatively printed on the PCL layers (Figure 6(A-I)). Spatial patterning of dual stem cells with dECM mimics the native tissue microenvironment and enhances cell-to-cell interactions, differentiation capability, vascularization, and tissue regeneration through the WT1-mediated Wnt/β-catenin signaling. In addition, the porcine heart dECM (bioink I) improves the maturation of CPCs compared to pure collagen (Figure 6(A-II)), while the porcine heart dECM (bioink II) promotes vascular formation (Figure 6(A-III)). What’s more, *in vivo* implantation of this pre-vascularized cardiac patch demonstrated therapeutic effects including the formation of neomuscle and capillaries at the infarct area and improvement of cardiac function.^209^ Another interesting study by Noor et al. indicated the potential of 3D bioprinting of the microvascular cardiac patch for revascularization and repair in the infarct area.^211^ They designed a model considering oxygen diffusion based on Fick’s second law and consumption based on Michaelis–Menten formula,^212^ to achieve optimum size, distribution, and orientation of the blood vessels (Figure 6(B-I)). For the 3D bioprinting, Omenta decellularized personalized bioink combined either with iPSC-CMs or neonatal cardiac cells was employed as bioinks in combination with gelatin sacrificial layer containing endothelial cells (ECs) to print the designed cardiac tissues with approximately 2 mm thickness (Figure 6(B-II and B-III)) and high cell viability (Figure 6(B-IV)) After incubation at 37°C, the ECs cells were attached to the edges of the dECM hydrogels, while the sacrificial bioink was washed away creating ≈300 μm diameter open blood vessels inside the patch (Figure 6(B-V and B-VII)). They were also able to fabricate miniature human hearts with main cellular lumens using volumetric printing in a support medium composed of alginate microparticles in xanthan gum-supplemented growth medium (Figure 6(B-VIII–X)).^211^

These reports show the great prospective of 3D bioprinting and dECM as powerful tools for developing functional cardiac tissues. One of the key advantages of the 3D bioprinting approach is the design control over the organization of the vascular network and homogeneous distribution of cell populations in the printed scaffolds that can create a microvascular network that facilitates blood vessel formation.^193^ Despite all the efforts, current 3D bioprinting approaches for dECM hydrogels still face some challenges and limitations such as low resolution and limitations in the fabrication of nanoscale components which calls for more investigations.

#### Electrospinning

To better mimic the distinct geometrical characteristics of the cardiac tissue at the nanoscale, electrospinning method has emerged as one of the attractive fabrication methods. It has been applied in the fabrication of biomimetic scaffolds with interconnected pores, high surface-to-volume ratio, and nanoscaled morphology.^213,214^ Electrospun scaffolds recapitulate the fibrillar architecture of the native ECM which is crucial for cell organization, survival, and function.^8^

Electrospun dECM-based scaffolds are known as promising platforms for cardiac tissue engineering due to the presence of essential cardiac ECM components and their fibrous structure. But unfortunately, they suffer from poor physical properties (such as mechanical properties) and spinnability.^215^ Earlier works have shown that hybrid strategies can enhance the cellular and mechanical properties of these scaffolds. For example, Reid et al. utilized aorta and heart ECM to create a scaffold with tailored biological and mechanical properties. These results demonstrated that the incorporation of aorta dECM into PCL compared to heart dECM generates a stiffer scaffold with better cell adhesion.^216^ Additionally, it can be considered a promising method for vascular tissue engineering applications. In another study, poly (ethylene oxide) (PEO) was added to the native decellularized porcine ECM to generate a spinnable dECM-based solution to enhance cardiac tissue engineering.^196^ Mashayekhan et al. have developed a fibrous-compatible hydrogel containing gelatin-PCL embedded in chitosan-dECM. This hybrid structure mimics the fibrillar architecture of the native cardiac tissue and its mechanical properties. In this approach, an ideal solvent such as HFIP was used to break hydrogen bonds and/or hydrophobic interactions to generate spinnable dECM-based solutions. It should be noted that applying this approach might not be practical due to HFIP’s toxicity.^217^ Additionally, the necessity of using a high electrical field during electrospinning may lead to the denaturation of dECM proteins.^218^ Another significant problem is the small average pore size of the nanofibers which leads to decreased cellular infiltration and cell migration into the electrospun mat. Therefore, a major problem of tissue-engineered constructs using electrospinning is the limited vascularization due to their dense fibrous structure.^219^

#### Soft lithography

The myocardium has a highly organized structure with a patterned micro/nanoscale anisotropic morphology. The myocardial microenvironment plays a vital role in the multitude of biological processes and regulating cellular behavior.^41,220,221^ Therefore, engineered constructs with controlled architectures and micro/nanoscale patterns can mimic the anisotropic nature of the myocardium and promote the alignment and maturation of cardiac cells.^222^ In this direction, soft lithography has provided a promising tool to fabricate micro/nanoscale anisotropic engineered cardiac tissue. Soft-lithography is a fast prototyping method.^223,224^ Using this method, microtissues, micropillars, microactuators, and microfluidic chips can be fabricated, and it provides a means of recapitulating complex architectures and biomimetic microenvironments for cells.^225,226^

Several recent studies have demonstrated that micro-scale grooves generated with soft lithography can considerably improve the expression of cardiac-specific genes and proteins, and improve the functionality of cardiac scaffolds through the alignment of cells.^227–229^ It has also been reported that the differentiation of stem cells can be directed by nanopatterning (nanopillars and nanosillons) fabricated by soft lithography, through controlling size and spacing of the nanostructures.^230–232^ In addition, the native myocardium is subjected to various mechanical and electrical cues that can play an important role in controlling the cell fate. Based on the literature, electrical stimulation significantly enhances cellular alignment, communication, and maturation. Furthermore, microactuators and microfluidic systems fabricated by standard soft lithographic techniques in PDMS enable the replication of mechanical, and electrical cues in the native tissue. This strategy has been successful in the improvement and maturation of cardiac tissue.^226^

## Conclusion and future perspectives

Nowadays, the decellularization techniques and recellularization strategies are widely used among scientists to produce dECM scaffolds that mimic the native tissue and promote cell adhesion, growth, proliferation, and differentiation. Specifically, dECM of myocardium tissue has attained remarkable attention for infarct cardiac repair. Bioactive cardiac dECM scaffolds provide a native-like microenvironment and biological signals to induce the reconstruction of damaged heart tissue when implanted in the body. Additionally, disease modeling and drug testing on *in vitro* dECM-based cardiac tissue models facilitate the prediction of more accurate responses *in vitro* and result in realistic and translational outcomes from preclinical studies.

Despite the great progress, there are several challenges that need to be addressed before cardiac dECM can be applied clinically. Current decellularization methods suffer from critical drawbacks. More specifically, chemical and mechanical approaches damage some essential biomechanical and biochemical properties that are critical for the inherent function of the dECM. Therefore, the combination of chemical, enzymatic, and mechanical techniques is required in order to achieve a balance in maintaining the biological agents. Such new techniques support and regulate the new tissue construction, and eliminate the components causing immunogenic responses. 

In addition to decellularization, dECM recellularization is another challenge that should be taken into account. Up to now, no seeding strategy has been approved optimal. The static cell seeding approach usually leads to intense cell density near the surface with inadequate penetration into the center of the cardiac dECM. This imperfect recellularization is formed due to heterogenous distribution and loss of cells during perfusion. The number of cells required for recellularization is another issue that should be considered besides the seeding process. High-quantity cardiomyocytes are usually attained by repopulating human-sized hearts which corresponds to about 4 billion specialized cardiomyocytes. However, the isolation and culture of a large number of cardiomyocytes might be challenging. In this case, exploiting iPSC-derived cells is a good substitute for the numerous cardiomyocytes required for cardiac tissue engineering. However, imperfect differentiations of iPSCs into cardiomyocytes would increment the secondary tumorigenic risks caused by contamination with non-cardiomyocytes and undifferentiated cells. As a result, the risk factors impacting iPSC’s tumor formation should be precisely evaluated.

Another noteworthy challenge for cardiac tissue engineering is associated with cardiac patches derived from dECM. These cardiac patches could induce arrhythmia exacerbating normal cardiac function. Consequently, surgical risk evaluations are mandatory before implementation in patients. Moreover, the safety and efficiency of dECM cardiac patches should be investigated more extensively. Currently, the majority of dECM patches are implanted in rodent animal models while few studies have been done on large animal models to determine the efficacy of decellularized heart ECM patches. Besides, long-term evaluation after implantation is necessary before clinical trials and commercialization.

The ideal tissue-engineered scaffold should emulate the architectures, physiochemical properties, and anisotropic mechanical and electrical properties of the native tissue. Novel fabrication methods such as 3D bioprinting, electrospinning, and soft lithography, have opened a new pathway toward fabricating functional scaffolds. However, each of these techniques still suffers from various limitations. The 3D bioprinting approach is limited by devices and bioinks which so far resulted in low printing resolution and inability to fabricate nanoscale structures. The advent of the electrospinning technique is hindered by the required high electrical field that can lead to dECM protein denaturation and small pore size that reduces cell migration and vascularization. Soft lithography has restrictions with hydrogel scaffolds having an ionic or chemical cross-linking ability. Thus, there is a need for further research on the equipments and more suitable biomaterials. All considered, cardiac dECM scaffolds are attractive and promising candidates for clinical studies once they can be further assessed in detail.

## Supplemental Material

Supplemental Material - Recent advances in soluble decellularized extracellular matrix for heart tissue engineering and organ modeling Click here for additional data file. Supplemental Material for Recent advances in soluble decellularized extracellular matrix for heart tissue engineering and organ modeling by Golara Kafili, Hannaneh Kabir, Amirhossein Jalali Kandeloos, Elham Golafshan, Sara Ghasemi, Shohreh Mashayekhan and Nayere Taebnia in Journal of Biomaterials Applications
