# Supplementary figures and images for: Recent advances in soluble decellularized extracellular matrix for heart tissue engineering and organ modeling
Source: J Biomater Appl. 2023 Nov 24;38(5):577–604. doi: 10.1177/08853282231207216 (PMC10676626; doi:10.1177/08853282231207216)

## Graphical Abstract

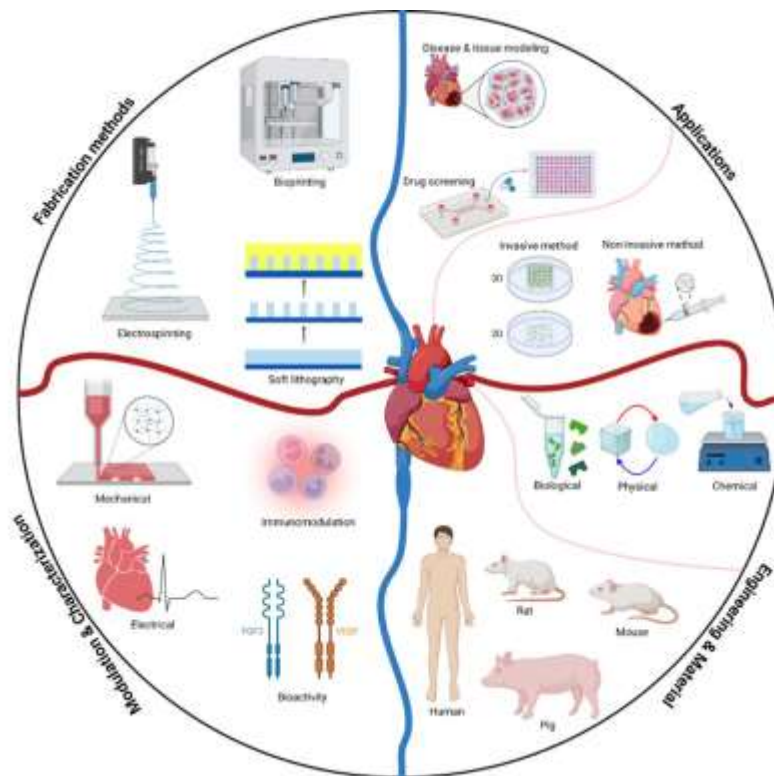

Supplement: Supplemental Material - Recent advances in soluble decellularized extracellular matrix for heart tissue engineering and organ modeling [file sj-pdf-1-jba-10.1177_08853282231207216.pdf]
